# Meta-analysis of Vascular Imaging Features to Predict Outcome Following Intravenous rtPA for Acute Ischemic Stroke

**DOI:** 10.3389/fneur.2016.00077

**Published:** 2016-05-18

**Authors:** Ricardo C. Nogueira, Edson Bor-Seng-Shu, Nazia P. Saeed, Manoel J. Teixeira, Ronney B. Panerai, Thompson G. Robinson

**Affiliations:** ^1^Department of Neurology, Hospital das Clínicas, University of São Paulo School of Medicine, São Paulo, Brazil; ^2^Department of Neurosurgery, Hospital das Clínicas, University of São Paulo School of Medicine, São Paulo, Brazil; ^3^Department of Cardiovascular Sciences, University of Leicester, Leicester, England; ^4^Biomedical Research Unit in Cardiovascular Science, Glenfield Hospital, Leicester, England

**Keywords:** ischemic stroke, clinical outcome, intracerebral hemorrhage, cerebral hemodynamics, intracranial circulation, cerebral autoregulation, rtPA

## Abstract

**Background:**

The present review investigated which findings in vascular imaging techniques can be used to predict clinical outcome and the risk of symptomatic intracerebral hemorrhage (sICH) in patients who underwent intravenous thrombolytic treatment.

**Methods:**

Publications were searched, and the inclusion criteria were as follows: (1) published manuscripts, (2) patients with acute ischemic stroke managed with intravenous recombinant tissue plasminogen activator (rtPA), and (3) availability of imaging assessment to determine vessel patency or the regulation of cerebral blood flow prior to, during, and/or after thrombolytic treatment. Clinical outcomes were divided into neurological outcome [National Institutes of Health Stroke Scale (NIHSS) within 7 days] and functional outcome (modified Rankin score in 2–3 months). sICH was defined as rtPA-related intracerebral bleeding associated with any worsening of NIHSS.

**Results:**

Thirty-nine articles were selected. Recanalization was associated with improved neurological and functional outcomes (OR = 7.83; 95% CI, 3.71–16.53; *p* < 0.001 and OR = 11.12; 95% CI, 5.85–21.14; *p* < 0.001, respectively). Both tandem internal carotid artery/middle cerebral artery (ICA/MCA) occlusions and isolated ICA occlusion had worse functional outcome than isolated MCA occlusion (OR = 0.26, 95% CI, 0.12–0.52; *p* < 0.001 and OR = 0.24, 95% CI, 0.07–0.77; *p* = 0.016, respectively). Reocclusion was associated with neurological deterioration (OR = 6.48, 95% CI, 3.64–11.56; *p* < 0.001), and early recanalization was associated with lower odds of sICH (OR = 0.36, 95% CI, 0.18–0.70; *p* = 0.003).

**Conclusion:**

Brain circulation data before, during, and after thrombolysis may be useful for predicting the clinical outcome. Cerebral arterial recanalization, presence and site of occlusion, and reocclusion are all important in predicting the clinical outcome.

## Introduction

A meta-analysis of recombinant tissue plasminogen activator (rtPA) trials suggested that there may be a group of patients who would benefit from thrombolysis within 6 h of symptom onset, if they were carefully selected using advanced imaging modalities (1).

There has been an extensive investigation of prognostic indices of good outcomes that can be applied before, during, and after thrombolysis (2). It is well known that factors, such as age, initial National Institutes of Health Stroke Scale (NIHSS) score, and systolic blood pressure, are of predictive value for clinical outcome and symptomatic intracerebral hemorrhage (sICH) (3). Magnetic resonance imaging (MRI), computed tomography (CT), and transcranial Doppler (TCD) have also been used as possible prognostic determinant tools (4). In particular, arterial occlusion, recanalization, and reocclusion, among other factors, have been investigated in terms of outcome prediction (4). However, there is a gap in the literature about how many methods, which evaluate intracranial circulation, have been used before and after thrombolysis to predict the outcome in a systematic fashion, together with a critical review of the strengths and weaknesses of each method.

The aim of the present review is (1) to undertake a descriptive systematic review of studies that have evaluated the intracranial circulation before, during, and after thrombolysis; (2) to evaluate the parameters that provide prognostic information of clinical outcome and/or sICH; and (3) to perform a meta-analysis of studies that used such parameters.

## Materials and Methods

### Search Strategy

A literature search strategy, restricted to publications from January 1994 to January 2015, was designed to identify cerebral hemodynamic studies, which assessed cerebral vessel patency and/or autoregulation in acute ischemic stroke patients treated with intravenous rtPA. Two reviewers (Ricardo C. Nogueira and Nazia P. Saeed) identified studies from PubMed database using the keywords “ischemic stroke” AND “rtPA treatment” OR “thrombolysis” AND “cerebral hemodynamics” OR “cerebral autoregulation” OR “cerebral blood flow control.” Bibliographic references of selected articles were examined for additional suitable studies. The inclusion criteria were (1) published manuscripts in English language, (2) patients (>18 years of age) with acute ischemic stroke treated with intravenous rtPA, and (3) availability of assessment of intracranial circulation to determine vessel patency and/or regulation of cerebral blood flow prior to, during, and/or after thrombolytic treatment. The exclusion criteria were (1) acute ischemic stroke not managed with intravenous rtPA, (2) impossibility to determine the vessel patency or the regulation of cerebral blood flow before, during, and/or after rtPA administration, (3) non-English language publications, (4) non-human models, and (5) case reports. The systematic review followed PRISMA guidelines; quality assessment of articles were used applying the American Academy of Neurology rating system by two raters (Ricardo C. Nogueira and Nazia P. Saeed), reviewed by a third investigator (Edson Bor-Seng-Shu) in terms of agreement, and this individual resolved any discrepancies. For the meta-analysis, authors of publications with incomplete data were contacted for additional information. Moreover, care was taken to exclude articles with no comparable data (for example: lack of imaging after thrombolysis, lack of clinical outcome measured, etc.) and articles with patients included in other articles from the same institution to avoid biasing the population sample.

### Data Extraction

The following data were extracted from each article: sample size, presence of a control group, type(s) of diagnostic modality used, time delay between assessment and thrombolysis (when stated), time interval between rtPA treatment and monitoring examinations (assessment during and following treatment, when stated), outcomes, conclusions, and study limitations. The articles were grouped according to the method of cerebral hemodynamic assessment. Data from cerebral hemodynamic assessment techniques were compared based on their common aspects, primary contributions, and limitations.

### Outcomes

Clinical outcomes were divided into functional, defined by modified Rankin scale (mRS) in the late post-thrombolytic therapy (2–3 months) with good outcome been considered as mRS ≤2, and neurological outcomes, assessed by NIHSS and comprising improvement (reduction of 10 points or final NIHSS ≤3) or deterioration (increase ≥4 points) in the early post-thrombolytic therapy stage (within 7 days). sICH was defined as rtPA-related intracerebral bleeding detected by CT or MRI associated with any worsening of NIHSS or death. Additional outcome measures were arterial recanalization assessed by different scales [thombolysis in brain ischemia (TIBI), thrombolysis in myocardial ischemia (TIMI), and partial or full recanalization], reocclusion (within 24 h), and cerebral infarct volume.

### Meta-analysis

The variables related to assessment of intracranial circulation were identified in each retrieved paper and, if they presented in the results a significant correlation with clinical outcome and sICH in a multivariate analysis, they were considered for the meta-analysis. The software used for meta-analysis was the OpenMetaAnalyst (Center for Evidence-based Medicine, Brown University School of Public Health – Providence, RI, USA), the binary random-effect method was applied, and the heterogeneity of studies was evaluated using *I*^2^ statistics. To identify for publication bias, a funnel plot and Egger’s test were applied in the analysis of any assessed variable, where information was available from three or more studies.

## Results

### Number of Studies Retrieved

**Figure 1:**
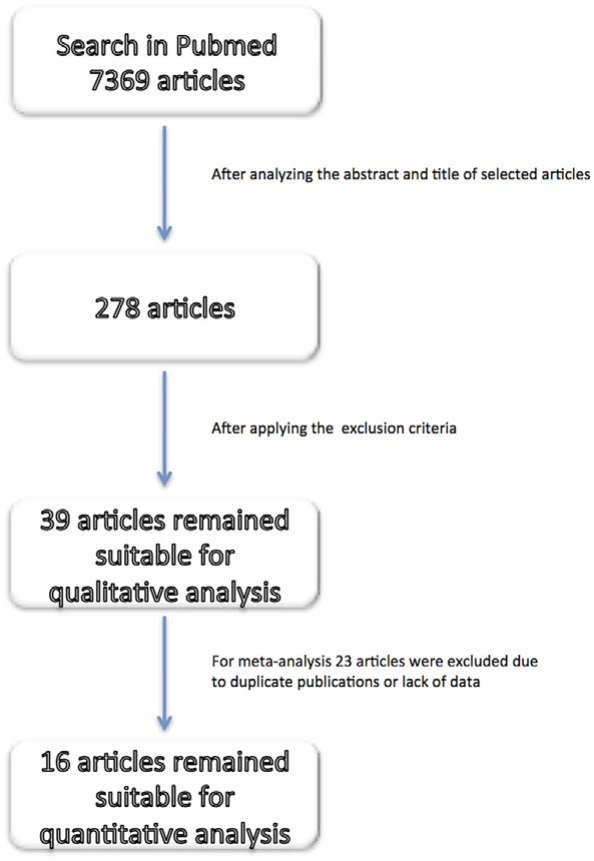
**Flow diagram of studies identification**.

The search in PubMed retrieved 7369 articles. After analyzing the title and abstract, a total of 278 articles were deemed suitable. The inclusion and exclusion criteria were then applied, leaving 39 articles for further analysis (Figure 1). Each article was then grouped according to the neuroimaging technique performed; 26 studies used TCD ultrasonography, 2 computed tomography angiography (CTA), 10 magnetic resonance imaging angiography (MRA), and 1 either MRA or CTA. TCD ultrasonography was the most used method for cerebral hemodynamic evaluation and was, on average, performed on more patients per study, besides presenting the shortest time interval for follow-up assessment (average 2.4 h).

### Characteristics of the Included Studies

There were 38 observational studies (37 cohort and 1 case-control) and 1 interventional study; no randomized controlled clinical trial was identified (please see Supplementary Material). The majority of the observational studies (36 of 38) used the neuroimaging methods for prognostic purposes, and the remainder used the methods for investigating diagnostic accuracy or causation.

After grouping the articles based on the type of cerebral hemodynamic assessment, common findings were as follows.

#### MR/CT Angiography

Thirteen studies using CTA (2 studies), MRA (10 studies), or MRA + CTA (1 study) were included (please see Supplementary Material) (5–17). In two studies (one using MRA and another CTA), neuroimaging assessment was not performed after rtPA treatment. The mean time delay from neuroimaging examination to thrombolytic therapy was 40 min, and the mean time for a second exam following therapy was 19.2 h. One study had an interventional design, and the main outcomes were functional and recanalization (TIMI 1–3). rtPA was associated with higher rates of recanalization and better functional outcomes. Concerning the observational studies, the findings revealed that the presence, site of arterial occlusion [proximal middle cerebral artery (MCA), distal MCA, or tandem internal carotid artery (ICA)–MCA], and the occurrence of partial or full recanalization were related to the clinical outcomes and final infarct size. The limitations included a small sample size and difficulties in determining the time of arterial recanalization.

#### Transcranial Doppler

Twenty-six studies used TCD as a method for cerebral hemodynamic assessment (please see Supplementary Material) (3, 18–42). In 24 studies, cerebral hemodynamic parameters were evaluated prior to and after thrombolytic treatment (range from 2 h to 2 days), while the remaining 2 studies presented only pre-rtPA cerebral hemodynamic data. TCD was the sole method for monitoring the cerebral hemodynamic status during thrombolysis.

These studies revealed that factors, such as pretreatment TIBI classification score, arterial recanalization/reocclusion, and the site of occlusion (tandem occlusions, proximal/distal ICA, or MCA occlusions), were associated with clinical outcome, infarct size, and sICH.

Limitations included the operator-dependence nature of the TCD method, the absence of arterial imaging resources (making it difficult to determine the site of occlusion), and unfavorable cranial windows for ultrasound energy passage.

### Pooled Results

The cerebral circulation variables associated with functional and neurological outcomes or sICH were arterial recanalization/reocclusion, presence/absence of arterial occlusion, and the site of occlusion (Tables 1 and 2). No article regarding cerebral autoregulation (CA) was identified.

**Table 1:** **Studies demonstrating association with clinical outcome**.

Article	Method	Variable	Outcome	Result
Molina et al. (19)	TCD	Early recanalization (6 h)	Good outcome (mRS ≤2) in 90 days	OR = 23.4 (5.4–96) *p* = 0.001
Nighoghossian et al. (12)	Multiparametric MRI	Recanalization	NIHSS at day 60	Multiple linear regression *p* = 0.0001
Molina et al. (3)	TCD	Proximal occlusion before thrombolysis	Good outcome (mRS ≤2) in 90 days	OR = 0.25 (0.10–0.61) *p* < 0.001
Sims et al. (17)	CTA	Absence of occlusion	Early improvement (4 points in NIHSS)	OR = 5.0 (1.1–23.3) *p* = 0.04
Sims et al. (17)	CTA	Absence of occlusion	Good outcome (mRS ≤2) in 7 days	OR = 6.8 (1.3–34.6) *p* = 0.02
Saqqur et al. (42)	TCD	Reocclusion	Clinical deterioration (4 points in NIHSS)	OR = 4.9 (1.7–13) *p* = 0.002
Saqqur et al. (41)	TCD	Distal × proximal MCA occlusion before thrombolysis	Good outcome (mRS ≤1) in 90 days	OR = 2.1 (1.1–4) *p* = 0.025
Tsivgoulis et al. (34)	TCD	Recanalization within 2 h	Good outcome (mRS ≤2) in 90 days	OR = 5.98 (2.58–13.84) *p* < 0.001

**Table 2:** **Studies demonstrating association with symptomatic intracerebral hemorrhage**.

Article	Method	Variable	Outcome	Result
Saqqur et al. (36)	TCD	Persistence of occlusion ≥2 h	sICH	OR = 6 (1.5–21.3) *p* = 0.01
Saqqur et al. (36)	TCD	Recanalization beyond 24 h or persistent occlusion	sICH	OR = 3 (1.1–10) *p* = 0.04
Saqqur et al. (36)	TCD	Persistence of proximal occlusion at 2 h	sICH	OR = 5 (1.5–15) *p* = 0.008
Saqqur et al. (36)	TCD	Persistence of proximal occlusion at 2 h (excluding reocclusion)	sICH	OR = 8 (3–26) *p* < 0.001

**Figure 2:**
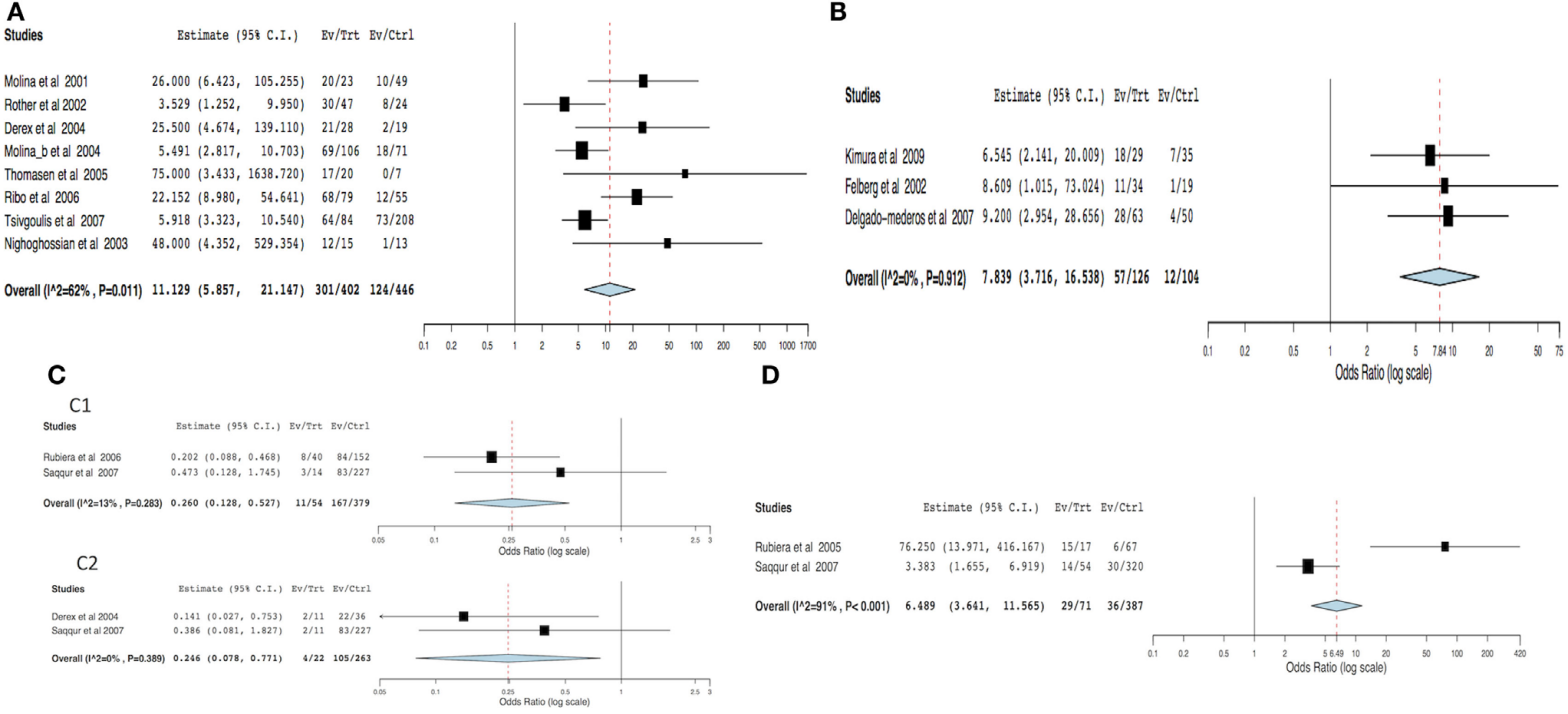
**Meta-analysis of hemodynamic variables related to clinical outcome**. **(A)** Functional outcome (dichotomized mRS) in recanalized versus non-recanalized patients. **(B)** Neurological outcome (NIHSS) in recanalized versus non-recanalized patients. **(C)** Functional outcome (dichotomized mRS) by site of occlusion: **(C1)** tandem ICA–MCA versus isolated MCA and **(C2)** isolated ICA versus isolated MCA. **(D)** Clinical deterioration in recanalization versus reocclusion patients. MCA – middle cerebral artery, ICA – internal carotid artery, NIHSS – National Institute of Health Stroke Scale, and mRS – modified Rankin scale.

**Figure 3:**
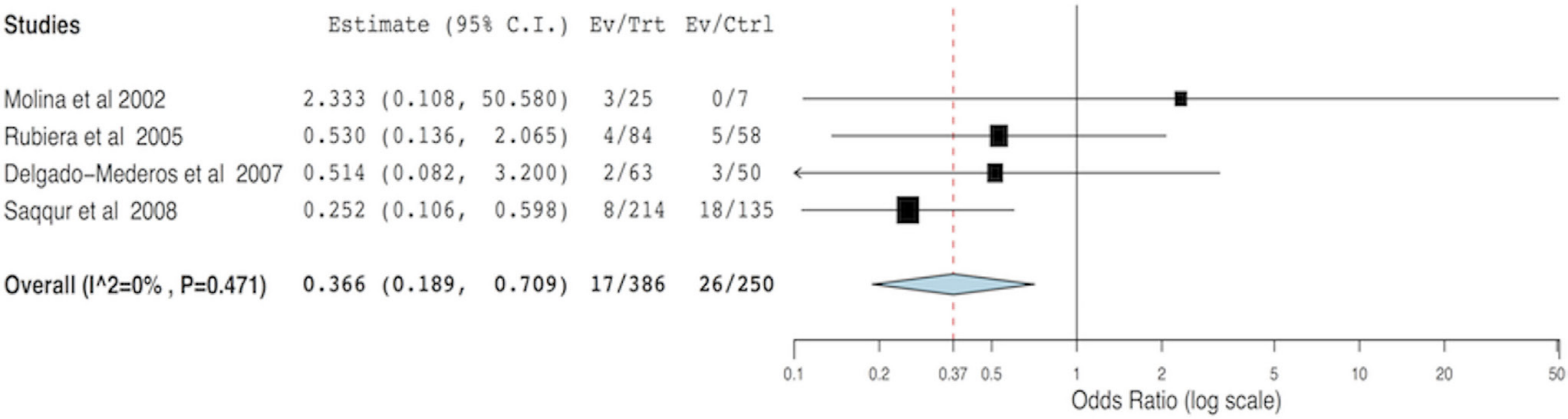
**Meta-analysis of the hemodynamic variables recanalization versus non-recanalization related to symptomatic intracerebral hemorrhage**.

For the meta-analysis, 23 out of 39 articles were excluded for the following reasons: possibility of overlapping patients (12 articles, 964 patients), lack of data able to be compared (6 articles, 360 patients), and lack of success in obtaining additional data (5 articles, 129 patients). Arterial recanalization was significantly associated with good functional outcome (OR = 11.12; 95% CI, 5.85–21.14; *p* < 0.001) and neurological improvement (OR = 7.83; 95% CI, 3.71–16.53; *p* < 0.001) (Figures 2A,B). Both tandem ICA/MCA occlusions and isolated ICA occlusion had worse functional outcome than isolated MCA occlusion (OR = 0.26, 95% CI, 0.12–0.52; *p* < 0.001 and OR = 0.24, 95% CI, 0.07–0.77; *p* = 0.016, respectively) (Figures 2C1,C2). Patients with recanalization followed by reocclusion within 24 h of thrombolysis had significant association with neurological deterioration (overall OR: 6.48, 95% CI: 3.64–11.56, *p* < 0.001) (Figure 2D). Early recanalization (up to 2 h after thrombolysis) was associated with lower odds of sICH (overall OR = 0.36, 95% CI, 0.18–0.70; *p* = 0.003) (Figure 3).

**Figure 4:**
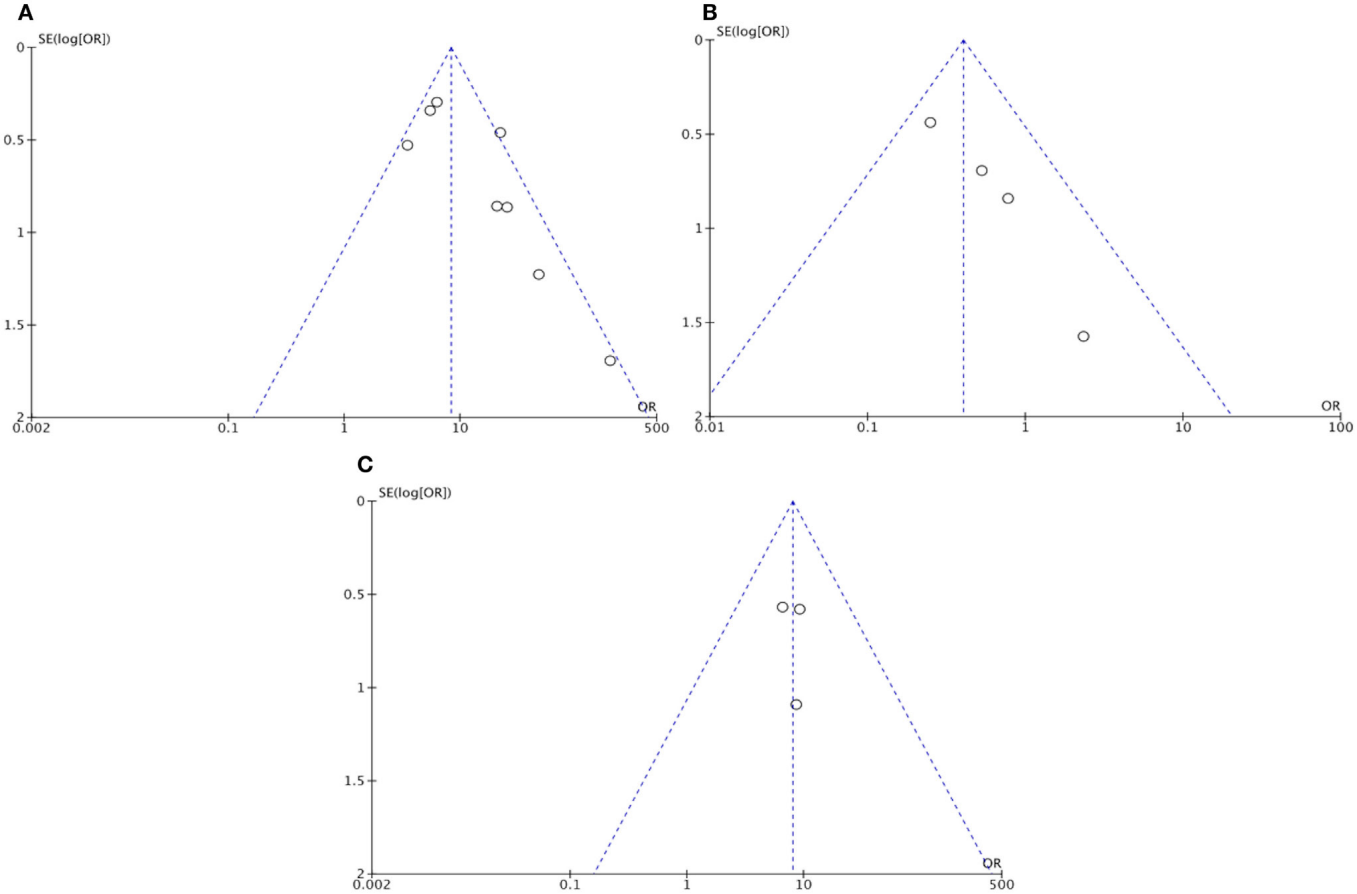
**Funnel plot assessing publication bias: (A) Functional outcome (dichotomized mRS) in recanalized versus non-recanalized patients; (B) Symptomatic intracranial hemorrhage in recanalization versus non-recanalization; and (C) Neurological outcome (NIHSS) in recanalized versus non-recanalized patients**. NIHSS – National Institute of Health Stroke Scale and mRS – modified Rankin scale.

The funnel plots of functional outcomes and sICH were suggestive of potential publication bias, which was confirmed by the Egger’s test (intercept: 43.65, *p* = 0.046; intercept: 1.97, *p* = 0.003; Figures 4A,B, respectively). However, the funnel plot of neurological improvement did not indicate the publication bias (Egger’s test intercept: 1.48, *p* = 0.83; Figure 4C).

## Discussion

In 2003, Schellinger et al. (43) highlighted the importance of various diagnostic modalities, such as brain MRI, CT, and TCD ultrasonography, for the decision-making process regarding thrombolytic therapy for acute ischemic stroke. Furthermore, the assessment of cerebral hemodynamic during and after thrombolytic therapy may be important to determine the parameters that could influence the clinical outcome, especially in the face of new research investigating the aggressive control of ABP in this scenario (44). Finally, with the new guidelines regarding endovascular treatment (45), the evaluation of cerebral circulation will be useful to determine proximal occlusion and also could select patients who were excluded from endovascular trials, but could benefit from the interventional therapy. To our knowledge, the present paper is the first to use techniques of systematic review and meta-analysis to verify the influence of assessment of intracranial circulation, provided by brain MRI, CT, and TCD ultrasonography on clinical outcomes and sICH in acute ischemic stroke patients who underwent thrombolytic therapy (46).

Considering the hemodynamic studies reviewed here, the specific details of each study are worth emphasizing. MRA and CTA represent valuable options for detecting and confirming large vessel occlusion, but cannot identify the timing of recanalization (8, 11, 15, 16, 37, 47–49). Furthermore, early imaging changes are the strong predictors of sICH (50) and could be used in association with cerebral circulation parameters. The primary limitations of MRA and CTA studies were the time delay in assessment after thrombolytic treatment, the use of intravenous contrast media, and the difficulties in determining the timing of recanalization (6, 9, 12, 48, 49). However, it is well established that the presence and timing of brain arterial recanalization correlates with clinical outcome, hemorrhagic complications, and infarct size (14, 19–22, 25, 29, 31, 36, 37). For this purpose, TCD ultrasonography remains a valuable choice, as this method exhibits the following advantages: (1) device portability, (2) low cost of the method, and (3) real-time monitoring during thrombolysis (3, 18, 20–23, 25, 26, 30, 34, 36, 37). TCD ultrasound may also play a therapeutic role during thrombolysis; a recent systematic review revealing that sonothrombolysis associated with rtPA is a safe procedure that increases recanalization rate in the acute ischemic stroke setting (51, 52). However, TCD is operator-dependent, cannot reliably monitor small brain artery occlusion, and does not determine the exact site of occlusion. Therefore, in the future, a combination of methods may be important.

Only variables that, in the retrieved papers, presented significant correlation with outcome (clinical or sICH) in a multivariate analysis were included in the present review. The main variables associated with clinical outcome or sICH were recanalization, reocclusion, and the presence and site of occlusion. Our meta-analysis showed that recanalization is significantly associated with functional and neurological outcomes, but not with sICH. In fact, some studies have shown that the time of recanalization is associated with neurological recovery (21), and the persistence of occlusion is associated to sICH (36). Our findings are in agreement with a previous meta-analysis, which found significant association between recanalization and clinical outcome (OR: 4.43) and no association between recanalization and sICH, but comparisons should be made with caution because the previous meta-analysis evaluated intravenous, intra-arterial, and mechanical therapies (53).

Most of the conclusions presented in this paper have already been presented in endovascular therapy (EVT) trials. However, some issues, such as reocclusion, are more suitable for investigation by non-invasive imaging techniques. Furthermore, we believe that the information gathered in this paper, reaffirming the conclusions obtained with EVT trials and pointing the positive and negative aspects of other imaging techniques, is important to support the utilization of multimodality imaging in stroke care. A multimodality approach may provide a better understanding of the natural evolution of this pathology, and perhaps better future selection of patients for interventional therapies, who are currently excluded. Finally, strengthening the importance of different imaging modalities in the intravenous therapy setting is important for centers that still do not have access to interventional therapy, especially in low-income countries.

There are few studies concerning CA during acute and subacute ischemic stroke. Existing publications on CA in ischemic stroke reveal a transient impairment of CA during the subacute stages of major ischemic stroke (54–57). Interestingly, using animal models, it has been demonstrated that rtPA displays neurotoxic properties that can disrupt the blood–brain barrier, damage vessels, and possibly impair the CA (58). To our knowledge, only one study of CA after rtPA treatment in humans is available, which concluded that this treatment does not contribute to impaired CA. However, this study evaluated CA 10–20 h after rtPA treatment; not eliminating the possibility of an initial detrimental effect by rtPA on CA (59).

The limitations of the current review are (1) the search strategy was restricted to just one database, although PubMed comprises a majority of the relevant articles on this topic; (2) the inclusion criteria were limited to research of standard dose of rtPA (0.9 mg/kg) for thrombolysis, although low dose of rtPA (0.6 mg/kg), other thrombolytic drugs (desmoteplase or tenecteplase), or other methods (mechanical thrombectomy, intra-arterial rtPA) have been investigated; (3) lack of analysis concerning the effect of recanalization time on clinical outcome, although the present meta-analysis showed the clinical impact of recanalization at the first 24 h; (4) use of different imaging modalities for detecting arterial occlusion and recanalization; (5) data heterogeneity (especially number of patients included in each study, quality of study, and average stroke severity) from different publications; and (6) potential publication bias of some outcomes’ measures confirmed by the funnel plot and Egger’s test.

## Conclusion

In conclusion, the use of brain assessments of cerebral circulation before, during, and after rtPA thrombolysis is promising, especially, to predict outcome. Arterial recanalization, presence and site of occlusion, and reocclusion relate to clinical outcome. Future studies of prognostic accuracy should investigate these factors, ideally using more than one method of cerebral hemodynamic assessment. In addition, the evaluation of cerebral blood flow regulation mechanisms during and after rtPA treatment should be explored.

## Author Contributions

RN contributed to the conception and design of research; acquisition, analysis, and interpretation of data; statistical analysis; drafting of the manuscript; and approved the final version. EB-S-S and NS contributed to the conception and design; interpretation of data; drafting of the manuscript; and approved the final version. MT, RP, and TR contributed to the analysis and interpretation of data; critical revision of the manuscript for important intellectual content; and approved the final version of the manuscript.

## Conflict of Interest Statement

The authors declare that the research was conducted in the absence of any commercial or financial relationships that could be construed as a potential conflict of interest.
