# Supplementary material for: Meta-analysis of Vascular Imaging Features to Predict Outcome Following Intravenous rtPA for Acute Ischemic Stroke
Source: Front Neurol. 2016 May 18;7:77. doi: 10.3389/fneur.2016.00077 (PMC4870283; doi:10.3389/fneur.2016.00077)
Supplement: Supplementary file 1 [file Table_1.DOCX]

**Supplementary Material**

**Meta-analysis of cerebral hemodynamic parameters to predict outcome following intravenous rtPA for acute ischemic stroke**

**Ricardo C Nogueira^1^, Edson Bor-Seng-Shu^2^, Nazia P Saeed^3^, Manoel J Teixeira^2^, Ronney B Panerai^3,4^, Thompson G Robinson^3,4^.**

^1^Department of Neurology, Hospital das Clinicas, University of São Paulo School of Medicine, São Paulo, Brazil

^2^Department of Neurosurgery, Hospital das Clinicas, University of São Paulo School of Medicine, São Paulo, Brazil

^3^ Department of Cardiovascular Sciences, University of Leicester, Robert Kilpatrick Clinical Sciences Building, PO Box 65, Leicester, England, LE2 7LX

^4^ Biomedical Research Unit in Cardiovascular Science, Glenfield Hospital, Leicester, LE3 9QP.

*Corresponding author:* RC Nogueira

Tel. +551126616401

e-mail: rcnogueira28@gmail.com

**Supplementary table.**

Summary of each hemodynamic assessment and its primary conclusions.

| **Article** | **Method** | **Number of patients** | **AAN**  **Score** | **Main conclusion** |
| --- | --- | --- | --- | --- |
| Linfante et al (2002) (1) | MR or CT angiography and TCD | 36 | IV | MCA occlusions are associated with lower NIHSS scores and higher proportion of recanalization when compared with ICA occlusions. |
| Röther et al (2002) (2) | MR angiography | 139 | III | rtPA therapy is associated with better vessel recanalization and functional outcome. |
| Nighoghossian et al (2003) (3) | MR angiography | 29 | II | Recanalization (day one) is correlated with clinical outcome after rtPA for ischemic stroke. |
| Derex et al (2004) (4) | MR angiography | 49 | II | The site of occlusion in pre-treatment MRA is associated with recanalization rate and clinical outcome. |
| Alsop et al (2005) (5) | MR angiography and TCD | 20 | IV | Extremely low or completely absent contrast arrival may indicate tissue-at-risk for hemorrhage before rtPA treatment. |
| Pialat et al (2005) (6) | MR angiography | 42 | IV | Recanalization is highly correlated with time from stroke onset to the thrombolytic treatment and seems to decrease the growth potential of the lesion. |
| Albers et al (2006) (7) | MR angiography | 74 | II | Early recanalization is associated with a higher reduction in PWI volume and better clinical outcome in mismatch patients. |
| Olivot et al (2008) (8) | MR angiography | 74 | III | Early recanalization is associated with reduced infarct growth and better clinical outcomes in mismatch patients, but not in the absence of mismatch |
| Olivot et al (2009) (9) | MR angiography | 32 | III | Early recanalization is associated with higher 30-day diffusion reversal rates |
| Kimura et al (2009) (10) | MR angiography | 64 | I | rtPA treatment is related to higher early recanalization rate in patients with major artery occlusion (MCA higher than ICA). |
| Baizabal-Carvallo (2012) (11) | MR angiography and TCD | 33 | III | Complete recanalization was independently associated with smaller infarct growth |
| Sims et al (2005) (12) | CT angiography | 47 | III | Patients without major artery occlusion before treatment have lower NIHSS, better chances of early improvement, fewer hemorrhages and early independence. |
| Sillanpaa et al (2012) (13) | CT angiography | 83 | II | Combination of angiogram with perfusion best predicts clinical outcome |
| Christou et al (2000) (14) | TCD | 40 | II | The timing of arterial recanalization after rtPA therapy as determined with TCD correlates with clinical recovery from stroke and demonstrates a 300-minute window to achieve early complete recovery. |
| Burgin et al (2000) (15) | TCD | 25 | II | Complete MCA recanalization on TCD accurately predicts angiographic findings. |
| Alexandrov et al (2000) (16) | TCD | 40 | II | Dramatic recovery during rtPA therapy was associated with recanalization on TCD, whereas no early improvement indicated persistent occlusion or re-occlusion. |
| Molina et al (2001) (17) | TCD | 72 | III | Early recanalization is a powerful independent predictor of functional independence at 3 months. |
| Alexandrov et al (2001) (18) | TCD | 65 | III | Rapid arterial recanalization is associated with better short-term improvement. |
| Alexandrov et al (2002) (19) | TCD | 60 | III | Early re-occlusion is responsible for neurologic deteriorations. However, patients with re-occlusion have better long-term outcomes than patients without any early recanalization. |
| Christou et al (2002) (20) | TCD and MR angiography or DSA | 20 | III | ICA occlusion is more resistant to recanalization after intravenous rtPA therapy. However, recanalization of associated proximal MCA clot or improved MCA collateral flow is strongly associated with good outcome. |
| Molina et al (2002) (21) | TCD | 32 | II | Thrombolysis-related HI (HI1-HI2) represents a marker of early successful recanalization, which leads to a reduced infarct size and improved clinical outcome. |
| Felberg et al (2002) (22) | TCD | 53 | II | TCD monitoring suggests that dramatic recovery is a result of early restoration of MCA flow during the rtPA infusion. |
| El-Mitwalli et al (2002) (23) | TCD | 95 | III | The number of collateral flow channels and Thrombolysis in Brain Ischemia (TIBI) flow grade is associated with NIHSS scores before thrombolysis. |
| Labiche et al (2003) (24) | TCD | 86 | IV | Patients with no detectable residual flow signals at TCD before thrombolysis, have less chance of complete early recanalization with intravenous rtPA. |
| Molina et al (2004) (25) | TCD | 72 | III | The pattern of rtPA-induced MCA recanalization differs among stroke subtypes. |
| Molina et al (2004) (26) | TCD | 177 | III | The combination of clinical, radiological, and hemodynamic information predicts with a high accuracy long-term stroke outcome during or shortly after intravenous rtPA administration. |
| Thomassen et al (2005) (27) | TCD | 41 | III | Recanalization within 24 h is associated with favorable outcome mostly within the first 5 h after stroke |
| Kim et al (2005) (28) | TCD | 104 | III | Tandem lesion has lower early recanalization rate and early neurological improvement than isolated MCA occlusion. |
| Rubiera et al (2005) (29) | TCD | 142 | III | Stroke severity and ipsilateral severe carotid artery disease independently predict re-occlusion after rtPA-induced MCA recanalization. |
| Rubiera et al (2006) (30) | TCD | 221 | I | Tandem occlusion of ICA/MCA independently predicts poor outcome after IV thrombolysis. |
| Ribo et al (2006) (31) | TCD | 179 | III | The majority of rtPA-induced recanalization occurs during the first hour after treatment. Late recanalization can still be associated with clinical improvement if achieved within 6 hours from onset. |
| Saqqur et al (2007) (32) | TCD | 374 | II | Early re-occlusion is predictive of clinical deterioration and long-term poor outcome. |
| Saqqur et al (2007_b) (33) | TCD | 335 | IV | Site of occlusion is associated with clinical response to thrombolysis. Terminal ICA occlusion is least likely to respond to treatment. |
| Tsivgoulis et al (2007) (34) | TCD | 351 | II | Higher pretreatment SBP levels are associated with poor recanalization in patients treated with intravenous rtPA. |
| Delgado-Mederos et al (2007) (35) | TCD and MRI | 113 | II | Time of recanalization predicts DWI lesion evolution and clinical outcome with slow recanalization being associated with greater DWI lesion growth and poorer short and long term outcomes |
| Saqqur et al (2008) (36) | TCD | 349 | II | Persistent arterial occlusion after intravenous rtPA treatment can be an independent predictor of SICH. |
| Delgado-Mederos et al (2008) (37) | TCD and MRI | 80 | II | Blood pressure variability in non-reanalyzed patients is associated with greater diffusion-weighted imaging lesion growth and worse clinical course. |
| Saqqur et al (2009) (38) | TCD | 361 | II | Pre-treatment TIBI flow grade predicts likelihood of complete recanalization, time of recanalization and long term outcome. |
| Uzuner ert al (2013) (39) | TCD | 90 | IV | Pulsatility index may be associated with clinical outcome after thrombolytic therapy |

**References**

1. Linfante I, Llinas RH, Selim M, Chaves C, Kumar S, Parker RA, et al. Clinical and vascular outcome in internal carotid artery versus middle cerebral artery occlusions after intravenous tissue plasminogen activator. *Stroke* (2002) **33**(8):2066-71. PubMed PMID: 12154264.

2. Rother J, Schellinger PD, Gass A, Siebler M, Villringer A, Fiebach JB, et al. Effect of intravenous thrombolysis on MRI parameters and functional outcome in acute stroke <6 hours. *Stroke* (2002) **33**(10):2438-45. PubMed PMID: 12364735.

3. Nighoghossian N, Hermier M, Adeleine P, Derex L, Dugor JF, Philippeau F, et al. Baseline magnetic resonance imaging parameters and stroke outcome in patients treated by intravenous tissue plasminogen activator. *Stroke* (2003) **34**(2):458-63. PubMed PMID: 12574560.

4. Derex L, Nighoghossian N, Hermier M, Adeleine P, Berthezene Y, Philippeau F, et al. Influence of pretreatment MRI parameters on clinical outcome, recanalization and infarct size in 49 stroke patients treated by intravenous tissue plasminogen activator. *J Neurol Sci* (2004) **225**(1-2):3-9. doi: 10.1016/j.jns.2004.05.020. PubMed PMID: 15465079.

5. Alsop DC, Makovetskaya E, Kumar S, Selim M, Schlaug G. Markedly reduced apparent blood volume on bolus contrast magnetic resonance imaging as a predictor of hemorrhage after thrombolytic therapy for acute ischemic stroke. *Stroke* (2005) **36**(4):746-50. doi: 10.1161/01.STR.0000158913.91058.93. PubMed PMID: 15746457.

6. Pialat JB, Wiart M, Nighoghossian N, Adeleine P, Derex L, Hermier M, et al. Evolution of lesion volume in acute stroke treated by intravenous t-PA. *J Magn Reson Imaging* (2005) **22**(1):23-8. doi: 10.1002/jmri.20363. PubMed PMID: 15971175.

7. Albers GW, Thijs VN, Wechsler L, Kemp S, Schlaug G, Skalabrin E, et al. Magnetic resonance imaging profiles predict clinical response to early reperfusion: the diffusion and perfusion imaging evaluation for understanding stroke evolution (DEFUSE) study. *Ann Neurol* (2006) **60**(5):508-17. doi: 10.1002/ana.20976. PubMed PMID: 17066483.

8. Olivot JM, Mlynash M, Thijs VN, Kemp S, Lansberg MG, Wechsler L, et al. Relationships between infarct growth, clinical outcome, and early recanalization in diffusion and perfusion imaging for understanding stroke evolution (DEFUSE). *Stroke* (2008) **39**(8):2257-63. doi: 10.1161/STROKEAHA.107.511535. PubMed PMID: 18566302; PubMed Central PMCID: PMC2706660.

9. Olivot JM, Mlynash M, Thijs VN, Purushotham A, Kemp S, Lansberg MG, et al. Relationships between cerebral perfusion and reversibility of acute diffusion lesions in DEFUSE: insights from RADAR. *Stroke* (2009) **40**(5):1692-7. Epub 2009/03/21. doi: 10.1161/STROKEAHA.108.538082

STROKEAHA.108.538082 [pii]. PubMed PMID: 19299632.

10. Kimura K, Iguchi Y, Shibazaki K, Aoki J, Uemura J. Early recanalization rate of major occluded brain arteries after intravenous tissue plasminogen activator therapy using serial magnetic resonance angiography studies. *Eur Neurol* (2009) **62**(5):287-92. Epub 2009/08/29. doi: 10.1159/000235753

000235753 [pii]. PubMed PMID: 19713704.

11. Baizabal-Carvallo JF, Rosso C, Alonso-Juarez M, Pires C, Samson Y. The hemodynamic status within 24 h after intravenous thrombolysis predicts infarct growth in acute ischemic stroke. *J Neurol* (2012) **259**(6):1045-50. Epub 2011/11/08. doi: 10.1007/s00415-011-6295-3. PubMed PMID: 22057406.

12. Sims JR, Rordorf G, Smith EE, Koroshetz WJ, Lev MH, Buonanno F, et al. Arterial occlusion revealed by CT angiography predicts NIH stroke score and acute outcomes after IV tPA treatment. *AJNR Am J Neuroradiol* (2005) **26**(2):246-51. Epub 2005/02/15. doi: 26/2/246 [pii]. PubMed PMID: 15709120.

13. Sillanpaa N, Saarinen JT, Rusanen H, Hakomaki J, Lahteela A, Numminen H, et al. The clot burden score, the Boston Acute Stroke Imaging Scale, the cerebral blood volume ASPECTS, and two novel imaging parameters in the prediction of clinical outcome of ischemic stroke patients receiving intravenous thrombolytic therapy. *Neuroradiology* (2012) **54**(7):663-72. Epub 2011/09/10. doi: 10.1007/s00234-011-0954-z. PubMed PMID: 21904832.

14. Christou I, Alexandrov AV, Burgin WS, Wojner AW, Felberg RA, Malkoff M, et al. Timing of recanalization after tissue plasminogen activator therapy determined by transcranial doppler correlates with clinical recovery from ischemic stroke. *Stroke* (2000) **31**(8):1812-6. Epub 2000/08/06. PubMed PMID: 10926939.

15. Burgin WS, Malkoff M, Felberg RA, Demchuk AM, Christou I, Grotta JC, et al. Transcranial doppler ultrasound criteria for recanalization after thrombolysis for middle cerebral artery stroke. *Stroke* (2000) **31**(5):1128-32. Epub 2000/05/08. PubMed PMID: 10797176.

16. Alexandrov AV, Demchuk AM, Felberg RA, Christou I, Barber PA, Burgin WS, et al. High rate of complete recanalization and dramatic clinical recovery during tPA infusion when continuously monitored with 2-MHz transcranial doppler monitoring. *Stroke* (2000) **31**(3):610-4. Epub 2000/03/04. PubMed PMID: 10700493.

17. Molina CA, Montaner J, Abilleira S, Arenillas JF, Ribo M, Huertas R, et al. Time course of tissue plasminogen activator-induced recanalization in acute cardioembolic stroke: a case-control study. *Stroke* (2001) **32**(12):2821-7. Epub 2001/12/12. PubMed PMID: 11739980.

18. Alexandrov AV, Burgin WS, Demchuk AM, El-Mitwalli A, Grotta JC. Speed of intracranial clot lysis with intravenous tissue plasminogen activator therapy: sonographic classification and short-term improvement. *Circulation* (2001) **103**(24):2897-902. Epub 2001/06/20. PubMed PMID: 11413077.

19. Alexandrov AV, Grotta JC. Arterial reocclusion in stroke patients treated with intravenous tissue plasminogen activator. *Neurology* (2002) **59**(6):862-7. Epub 2002/09/26. PubMed PMID: 12297567.

20. Christou I, Felberg RA, Demchuk AM, Burgin WS, Malkoff M, Grotta JC, et al. Intravenous tissue plasminogen activator and flow improvement in acute ischemic stroke patients with internal carotid artery occlusion. *J Neuroimaging* (2002) **12**(2):119-23. Epub 2002/04/30. PubMed PMID: 11977905.

21. Molina CA, Alvarez-Sabin J, Montaner J, Abilleira S, Arenillas JF, Coscojuela P, et al. Thrombolysis-related hemorrhagic infarction: a marker of early reperfusion, reduced infarct size, and improved outcome in patients with proximal middle cerebral artery occlusion. *Stroke* (2002) **33**(6):1551-6. Epub 2002/06/08. PubMed PMID: 12052990.

22. Felberg RA, Okon NJ, El-Mitwalli A, Burgin WS, Grotta JC, Alexandrov AV. Early dramatic recovery during intravenous tissue plasminogen activator infusion: clinical pattern and outcome in acute middle cerebral artery stroke. *Stroke* (2002) **33**(5):1301-7. Epub 2002/05/04. PubMed PMID: 11988607.

23. El-Mitwalli A, Saad M, Christou I, Malkoff M, Alexandrov AV. Clinical and sonographic patterns of tandem internal carotid artery/middle cerebral artery occlusion in tissue plasminogen activator-treated patients. *Stroke* (2002) **33**(1):99-102. Epub 2002/01/10. PubMed PMID: 11779896.

24. Labiche LA, Malkoff M, Alexandrov AV. Residual flow signals predict complete recanalization in stroke patients treated with TPA. *J Neuroimaging* (2003) **13**(1):28-33. Epub 2003/02/21. PubMed PMID: 12593128.

25. Molina CA, Montaner J, Arenillas JF, Ribo M, Rubiera M, Alvarez-Sabin J. Differential pattern of tissue plasminogen activator-induced proximal middle cerebral artery recanalization among stroke subtypes. *Stroke* (2004) **35**(2):486-90. Epub 2004/01/07. doi: 10.1161/01.STR.0000110219.67054.BF

01.STR.0000110219.67054.BF [pii]. PubMed PMID: 14707233.

26. Molina CA, Alexandrov AV, Demchuk AM, Saqqur M, Uchino K, Alvarez-Sabin J, et al. Improving the predictive accuracy of recanalization on stroke outcome in patients treated with tissue plasminogen activator. *Stroke* (2004) **35**(1):151-6. Epub 2003/12/13. doi: 10.1161/01.STR.0000106485.04500.4A

01.STR.0000106485.04500.4A [pii]. PubMed PMID: 14671245.

27. Thomassen L, Waje-Andreassen U, Naess H, Aarseth J, Russell D. Doppler ultrasound and clinical findings in patients with acute ischemic stroke treated with intravenous thrombolysis. *Eur J Neurol* (2005) **12**(6):462-5. Epub 2005/05/12. doi: ENE1008 [pii]

10.1111/j.1468-1331.2005.01008.x. PubMed PMID: 15885051.

28. Kim YS, Garami Z, Mikulik R, Molina CA, Alexandrov AV, Collaborators C. Early recanalization rates and clinical outcomes in patients with tandem internal carotid artery/middle cerebral artery occlusion and isolated middle cerebral artery occlusion. *Stroke* (2005) **36**(4):869-71. Epub 2005/03/05. doi: 01.STR.0000160007.57787.4c [pii]

10.1161/01.STR.0000160007.57787.4c. PubMed PMID: 15746449.

29. Rubiera M, Alvarez-Sabin J, Ribo M, Montaner J, Santamarina E, Arenillas JF, et al. Predictors of early arterial reocclusion after tissue plasminogen activator-induced recanalization in acute ischemic stroke. *Stroke* (2005) **36**(7):1452-6. Epub 2005/06/11. doi: 01.STR.0000170711.43405.81 [pii]

10.1161/01.STR.0000170711.43405.81. PubMed PMID: 15947260.

30. Rubiera M, Ribo M, Delgado-Mederos R, Santamarina E, Delgado P, Montaner J, et al. Tandem internal carotid artery/middle cerebral artery occlusion: an independent predictor of poor outcome after systemic thrombolysis. *Stroke* (2006) **37**(9):2301-5. Epub 2006/08/05. doi: 10.1161/01.STR.0000237070.80133.1d. PubMed PMID: 16888266.

31. Ribo M, Alvarez-Sabin J, Montaner J, Romero F, Delgado P, Rubiera M, et al. Temporal profile of recanalization after intravenous tissue plasminogen activator: selecting patients for rescue reperfusion techniques. *Stroke* (2006) **37**(4):1000-4. Epub 2006/03/04. doi: 01.STR.0000206443.96112.d9 [pii]

10.1161/01.STR.0000206443.96112.d9. PubMed PMID: 16514102.

32. Saqqur M, Molina CA, Salam A, Siddiqui M, Ribo M, Uchino K, et al. Clinical deterioration after intravenous recombinant tissue plasminogen activator treatment: a multicenter transcranial Doppler study. *Stroke* (2007) **38**(1):69-74. Epub 2006/12/02. doi: 01.STR.0000251800.01964.f6 [pii]

10.1161/01.STR.0000251800.01964.f6. PubMed PMID: 17138949.

33. Saqqur M, Uchino K, Demchuk AM, Molina CA, Garami Z, Calleja S, et al. Site of arterial occlusion identified by transcranial Doppler predicts the response to intravenous thrombolysis for stroke. *Stroke* (2007) **38**(3):948-54. Epub 2007/02/10. doi: 01.STR.0000257304.21967.ba [pii]

10.1161/01.STR.0000257304.21967.ba. PubMed PMID: 17290031.

34. Tsivgoulis G, Saqqur M, Sharma VK, Lao AY, Hill MD, Alexandrov AV. Association of pretreatment blood pressure with tissue plasminogen activator-induced arterial recanalization in acute ischemic stroke. *Stroke* (2007) **38**(3):961-6. Epub 2007/01/27. doi: 10.1161/01.STR.0000257314.74853.2b. PubMed PMID: 17255548.

35. Delgado-Mederos R, Rovira A, Alvarez-Sabin J, Ribo M, Munuera J, Rubiera M, et al. Speed of tPA-induced clot lysis predicts DWI lesion evolution in acute stroke. *Stroke* (2007) **38**(3):955-60. Epub 2007/02/10. doi: 01.STR.0000257977.32525.6e [pii]

10.1161/01.STR.0000257977.32525.6e. PubMed PMID: 17290028.

36. Saqqur M, Tsivgoulis G, Molina CA, Demchuk AM, Siddiqui M, Alvarez-Sabin J, et al. Symptomatic intracerebral hemorrhage and recanalization after IV rt-PA: a multicenter study. *Neurology* (2008) **71**(17):1304-12. Epub 2008/08/30. doi: 10.1212/01.wnl.0000313936.15842.0d. PubMed PMID: 18753474.

37. Delgado-Mederos R, Ribo M, Rovira A, Rubiera M, Munuera J, Santamarina E, et al. Prognostic significance of blood pressure variability after thrombolysis in acute stroke. *Neurology* (2008) **71**(8):552-8. Epub 2008/06/14. doi: 10.1212/01.wnl.0000318294.36223.69. PubMed PMID: 18550860.

38. Saqqur M, Tsivgoulis G, Molina CA, Demchuk AM, Shuaib A, Alexandrov AV, et al. Residual flow at the site of intracranial occlusion on transcranial Doppler predicts response to intravenous thrombolysis: a multi-center study. *Cerebrovasc Dis* (2009) **27**(1):5-12. Epub 2008/11/20. doi: 10.1159/000172628

000172628 [pii]. PubMed PMID: 19018132.

39. Uzuner N, Ozdemir O, Tekgol Uzuner G. Relationship between pulsatility index and clinical course of acute ischemic stroke after thrombolytic treatment. *Biomed Res Int* (2013) **2013**:265171. Epub 2013/08/29. doi: 10.1155/2013/265171. PubMed PMID: 23984332.
